# Serbia is in crisis

**DOI:** 10.1016/j.lanepe.2025.101413

**Published:** 2025-08-01

**Authors:** The Lancet Regional Health – Europe

In Serbia, lecture halls were vacated long before the academic year ended. Since November, 2024, thousands of students launched a nationwide university blockade, occupying campuses to demand justice, transparency, and democratic reform. The protests were triggered by the deadly collapse of a newly renovated train station canopy in the city of Novi Sad, which killed 16 people and became a symbol of the human cost of systemic corruption. Rather than investigating the tragedy, President Aleksandar Vučić’s Government escalated repression and portrayed student demonstrators as foreign-backed threats. The protest movement has drawn renewed attention to Vučić’s growing authoritarianism and the increasingly illiberal regime.

Despite facing tear gas and crowd-control weapons, including reportedly sonic weapons prohibited under Serbian law, the student-led movement has remained peaceful and persistent. Silent vigils are held countrywide at 1152 h every Friday, the moment the canopy collapsed. A group of students cycled from Novi Sad to Strasbourg; others completed a 1993 km ultramarathon relay to Brussels to deliver a letter to the European Parliament outlining their demands.

Over 3500 protests and assemblies were recorded in March and April 2025, and even though the movement has been termed the largest student-led mobilisation in Europe since 1968, the response from European leaders has been muted. This silence is increasingly difficult to justify, especially as the movement highlights the erosion of three pillars essential to any society: democracy, education, and health.

In the past decade, Serbia has seen the fifth-largest drop globally in the Liberal Democracy Index, published by Sweden's V-Dem Institute, ranking 41st out of 45 European countries. Serbia is also rated only partly free by Freedom House, a global civil liberties watchdog.

The protests reflect a deeper crisis: the erosion of public institutions meant to educate, protect, and care for the population. Among them, health care stands out as a powerful symbol of systemic decay.

Years of underfunding, political interferences in appointments, and widespread informal payments have left Serbia's health system fragile and distrusted. More than 70% of citizens believe the medical profession is corrupt. One in five patients admits to paying unofficially for care. Medical professionals are often appointed for their political ties, not qualifications. This culture of clientelism and opacity has bred inequality, undermined trust, and denied many of timely, effective treatment.

Meanwhile, political interference in health has escalated into the intimidation of health-care workers. Reports indicate government-linked officials pressured emergency doctors to withhold medical records from lawyers and human rights groups documenting protest-related injuries. The Assembly of Serbian Health Workers has called for an end to what it terms politically motivated disciplinary actions against staff who treated protesters. Together, these developments underscore how political interference reaches clinical care—where healing is treated as dissent.

All of this further compounds the challenges facing Serbia's already struggling health-care system. Serbia has one of the highest emigration rates among health-care workers in Europe, with just over 300 doctors per 100,000 people—among the lowest ratios in Europe. Low salaries and poor working conditions are often cited as key reasons for emigration, however, the recent protests point to a broader crisis of trust and morale—driven by political interference, little institutional support, and erosion of professional autonomy.

The protests have exposed the harsh realities of Serbia's health system: when systems are governed by political interests and fear rather than transparency and merit, equity, quality, and trust are impossible. The consequences are real—lives lost, dignity denied, and quite exile.

The student strikes in Serbia are not acts of defiance against the nation—they are demands for a better one. Students are calling for public institutions that are just, transparent, and accountable, and for systems that reward competence over political connections. These are democratic demands, rooted in the belief that governance should serve the public good. When institutions fail, health systems suffer. Corruption and the erosion of public trust directly compromise the delivery of care. Serbia is in crisis—and the cracks in its democracy are reflected in the failures of its health system.

Serbia stands at a crossroads. The student protests reflect a powerful call for transparency, accountability, and justice. If the Serbian Government continues to respond with repression and the international community remains silent, the erosion of democratic institutions, social trust, and public health could be irreversible. While hope remains for a future founded on these principles, achieving it will require a fundamental shift in political will within Serbia and meaningful engagement from the international community.

